# Infectious Disease Risk and Vaccination in Northern Syria after 5 Years of Civil War: The MSF Experience

**DOI:** 10.1371/currents.dis.bb5f22928e631dff9a80377309381feb

**Published:** 2018-02-02

**Authors:** Alan de Lima Pereira, Rosamund Southgate, Hikmet Ahmed, Penelope O’Connor, Vanessa Cramond, Annick Lenglet

**Affiliations:** Médecins Sans Frontières (MSF), Operational Centre Amsterdam (OCA), Kobanê, Syria; Médecins Sans Frontières (MSF), Kobanê, Syria; Public Health Department, Médecins Sans Frontières (MSF), Amsterdam, the Netherlands; Kobanê Health Administration (KHA), Kobanê, Syria; Médecins Sans Frontières (MSF), Kobanê, Syria; Public Health Department, Médecins Sans Frontières (MSF), Amsterdam, the Netherlands; Public Health Department, Médecins Sans Frontières, Operational Center Amsterdam, Amsterdam, The NetherlandsMedecins Sans Frontieres, Operational Centre Amsterdam

## Abstract

**Introduction:**

In 2015, following an influx of population into Kobanê in northern Syria, Médecins Sans Frontières (MSF) in collaboration with the Kobanê Health Administration (KHA) initiated primary healthcare activities. A vaccination coverage survey and vaccine-preventable disease (VPD) risk analysis were undertaken to clarify the VPD risk and vaccination needs. This was followed by a measles Supplementary Immunization Activity (SIA). We describe the methods and results used for this prioritisation activity around vaccination in Kobanê in 2015.

**Methods:**

We implemented a pre-SIA survey in 135 randomly-selected households in Kobanê using a vaccination history questionnaire for all children <5 years. We conducted a VPD Risk Analysis using MSF ‘Preventive Vaccination in Humanitarian Emergencies’ guidance to prioritize antigens with the highest public health threat for mass vaccination activities. A Measles SIA was then implemented and followed by vaccine coverage survey in 282 randomly-selected households targeting children <5 years.

**Results:**

The pre-SIA survey showed that 168/212 children (79.3%; 95%CI=72.7-84.6%) had received one vaccine or more in their lifetime. Forty-three children (20.3%; 95%CI: 15.1-26.6%) had received all vaccines due by their age; only one was <12 months old and this child had received all vaccinations outside of Syria. The VPD Risk Analysis prioritised measles, Haemophilus Influenza type B (Hib) and Pneumococcus vaccinations. In the measles SIA, 3410 children aged 6-59 months were vaccinated. The use of multiple small vaccination sites to reduce risks associated with crowds in this active conflict setting was noted as a lesson learnt. The post-SIA survey estimated 82% (95%CI: 76.9-85.9%; n=229/280) measles vaccination coverage in children 6-59 months.

**Discussion:**

As a result of the conflict in Syria, the progressive collapse of the health care system in Kobanê has resulted in low vaccine coverage rates, particularly in younger age groups. The repeated displacements of the population, attacks on health institutions and exodus of healthcare workers, challenge the resumption of routine immunization in this conflict setting and limit the use of SIAs to ensure sustainable immunity to VPDs. We have shown that the risk for several VPDs in Kobanê remains high.

**Conclusion:**

We call on all health actors and the international community to work towards re-establishment of routine immunisation activities as a priority to ensure that children who have had no access to vaccination in the last five years are adequately protected for VPDs as soon as possible.

## Introduction

The on-going, protracted conflict in Syria has led to a large scale breakdown of health services with a decrease in life expectancy and an increase in childhood mortality since the war began that has obliterated the public health gains being made in the past 1^,^^2^^,^^3^. Despite moderately high pre-conflict vaccine preventable disease (VPD) vaccination coverage rates in Syria 4 , recent reports of outbreaks of acute flaccid paralysis and measles have become increasingly common 5^,^^6^^,^^7^. While much has been documented about the health status of Syrians who are hosted as refugees in other countries, insufficient information is available about the effects of the conflict on the health of the population inside Syria 8 .

Kobanê (formerly Ayn al-Arab) is a town in Aleppo governorate in northern Syria. It participated in the full national Expanded Program of Immunization (EPI) prior to 2011. The implementation of EPI deteriorated as the conflict progressed and the programme was discontinued nationwide by mid-2014 9. Since then only some supplementary immunization activity (SIA) with oral polio vaccine was carried out by local authorities and supported by UNICEF.

The majority of the population of Kobanê fled the town at the end of 2014 following the takeover by the Islamic State (ISIS) with the majority of the 60,000 inhabitants taking refuge in neighbouring Turkey. In Turkey, some children re-started EPI as refugees. By the first half of 2015, people started returning to Kobanê as active fighting had subsided and as access improved, Médecins Sans Frontières (MSF) restarted healthcare activities in the region. A population census that was conducted by MSF and a local community-based organisation in May 2015 indicated that 24,000 people were living in Kobanê city at that time, across 4400 households.

In May 2015, continued population movement, limited access to health services and poor water and sanitation created ideal conditions for outbreaks of VPD amongst the returning population. Given the complete lack of routine vaccination, absence of recent coverage data and the risk of VPD transmission in this setting, MSF conducted a vaccine coverage survey in order to make an informed decision about prioritisation of public health interventions, particularly vaccination.

Based on the results of the survey and the VPD risk assessment that followed, measles SIA (i.e. a mass vaccination campaign) was carried out in Kobanê city and the surrounding county by the Kobanê Health Administration (KHA) with the support of MSF. This was followed by a post vaccination coverage survey to monitor the success of the SIA.

We describe the methodological processes and findings of the initial vaccination coverage survey, VPD risk analysis, mass measles vaccination program and subsequent post-campaign vaccination coverage survey in this conflict affected area. The information highlights the impact of the conflict on the vaccination status of Syrian children and the grave public health risks associated with this.

## Methodology

**Pre-SIA Vaccine Coverage Survey**

Target population and sample size

Children less than 5 years old (0-59 months) and living in Kobanê city at the time of the survey in June 2015 were included. The required sample size was calculated as 135 households, using ‘ENA for SMART’ software and the following assumptions: 17.1% of population of Kobanê city under 5 years old, 40% of children under 5 years fully vaccinated for their age, precision of 10%, design effect 1 (random sampling of households), average household size 5.4 and 10% non-response of households 10. The assumption of 40% coverage in the target population was based on the knowledge that the largest possible sample size for such a survey would be based on 50% coverage.

Sampling strategy

Simple random sampling was undertaken using Epop (Epicentre, Paris, France) software and Google Earth to generate 135 random geographic points within demarcated city limits. If the house nearest the point was not inhabited (many houses in Kobanê remained abandoned at this time), the next-nearest-front-door method was used to find another household. All children under 5 years in the selected households were included in the survey.

Data collection and analysis

The vaccination history questionnaire covered demographics of children under 5 in the house, vaccination history (verbal or from vaccination card and, for Bacille Calmette Guerin (BCG), presence or absence of BCG scar), and reasons for non‑vaccination (if not fully vaccinated). Verbal vaccination histories involved asking the care taker to describe each occasion that child had received vaccination(s), including age of child at the time, whether needle or drops were given, and into what limb(s).

Where the caretaker had no vaccination card and did not recall the name of vaccines given but the child had received all vaccines in Kobanê, a former local vaccination supervisor and a medical doctor compared the reported vaccination history with the EPI schedule previously used in Kobanê, and, where possible, determined what antigens were most likely to have been given. For instance, where a carer reported the child received vaccinations around 3, 4 and 5 months of age and involved two needles, it was assumed that these represented the pentavalent and inactivated polio vaccines on the Kobanê EPI schedule. A time lag of up to 1 month (before or after due date) for vaccinations due in the first year of life, and a time lag of up to 3 months for those vaccines due at 18 months was used as the range of tolerability. If reported vaccination episodes fell outside of these ranges then the antigen received in that vaccine episode was considered unidentifiable.

Parents/guardians of unimmunised or partially immunised children were asked the reason(s) for incomplete vaccination.

The World Health Organization (WHO) definition of a ‘fully immunized’ child was used to define full immunisation 11. Data collectors were daily workers from the town who all had university degrees and could speak Kurdish and Arabic (the local languages). They received two days of training on childhood vaccinations, sampling and data collection using the survey tools. They were supervised by the MSF public health liaison officer and the vaccination nurse and the team was led by a public health doctor who conducted intermittent field visits and was otherwise available over teleconferencing.

Data analysis was performed using Microsoft Excel and STATA (v.14.0). Vaccination coverage was calculated as the proportion of children that reported receiving the vaccine in the survey sample divided by the total number of children in the target vaccination age in the survey sample, with respective 95% confidence intervals (95%CI) calculated in STATA, taking into consideration the standard errors for that proportion.

**VPD Risk Analysis**

MSF Internal Guidelines ‘Preventative Vaccination in Emergencies’ and the WHO SAGE document ‘Vaccination in Acute Humanitarian Emergencies’ were used to prioritize antigens with the highest public health threat and guide decisions on vaccination activity 12^,^^13^.

The MSF risk analysis process involves examining six contextual risk factors in the target population (overcrowding; malnutrition; poor water, hygiene and sanitation systems; low access to health services; high birth rate; burden of HIV/AIDS/chronic disease) and grading each as: not a concern at the moment (grade 0), concerning (grade 1) or serious (grade 2). An automated scoring system then assigns a ‘spread likelihood’ score (from 0 to 8) for each of 12 VPDs for each contextual risk factor. This ‘spread likelihood’ score for each factor and disease combination represents the relative importance of the particular risk factor in promoting the spread of the particular disease. For instance, if a user assigns the factor ‘overcrowding’ the grade of 2 (serious), the tool gives measles a spread likelihood score of 8 for that factor (as serious overcrowding has high relevance for the spread of measles), and gives Hib a score of 4 (as serious overcrowding has a moderate relevance for the spread of Hib) and Yellow Fever a score of 2 (as serious overcrowding has a low relevance for the spread of Yellow Fever). The scores for each of the six spread factors for each disease are added together to give a total Spread Likelihood score for that disease in this context.

The scoring system was developed by the MSF International Working Group, based expert input plus the WHO SAGE’s ‘relevance of each general risk factor to each VPD’ scale published in its document ‘Vaccination in Acute Humanitarian Emergencies’.

The next step of the risk analysis is to look at aggravating epidemiological factors for each of the 12 VPDs, in terms of geographical or seasonal threats, population immunity and burden of the disease. Combining the results of these two steps produces a list of priority antigens to consider for preventative vaccination. To select those antigens that will be delivered in the context, a feasibility assessment is undertaken considering the window of opportunity for intervention, characteristics of each vaccine, target population, vaccination strategies, operational/logistical constraints and ethical issues.

**Measles SIA**

The KHA, with the support of MSF, aimed to vaccinate 95% of children aged 6-59 months in Kobanê city for measles (4560/4800 children). All children aged 6-59 months were offered measles vaccination unless a vaccination card showing two previous doses of measles or Measles Mumps Rubella (MMR) vaccine could be shown by the child’s parent/guardian. Each vaccinated child received a vaccination card and had their left index finger marked with an indelible ink pen for identification in the subsequent vaccination coverage survey. Skilled medical staff and support staff were identified by KHA and trained by MSF. Simultaneously, community sensitisation was conducted through printed pamphlets, meeting with various authorities, and messaging via loudspeakers, social media and door-to-door visits. Four geographically representative, public locations across Kobanê (schools and health centres) were chosen as vaccination sites. A 22m3 cold room powered by a generator was set up to store all the cold chain items. Tally sheets recording the number of vaccinations administered and vaccine vial consumption were used. Administrative vaccination coverage for the target population was calculated at the end of each day and at the end of the campaign using tally sheet data. Surveillance for Adverse Events Following Immunization (AEFI) was undertaken and a line list maintained.

**Post-SIA Vaccine Coverage Survey**

Target population and sample size

All those aged 6-59 months living in Kobanê city, Syria, during the time of the SIA were included in the target population. The required sample size was calculated as 282 households, using OpenEpi software and the following assumptions: 15% of population of Kobanê aged 6-59 months, 80% measles vaccination coverage from the campaign, precision of 5%, design effect of 1 and 20% non-response rate 14.

Sampling strategy

Similar to the vaccination coverage survey described earlier, we conducted simple random sampling with Epop mapping software to generate 282 random geographic points within demarcated city limits. If the house nearest the point was not inhabited the next-nearest-front-door method was used to find another household.

Data collection and analysis

A standardized questionnaire was used to collect the following data for each child: demographic data (age and sex), vaccination status for the Measles SIA (confirmed verbally by caretaker and/or by vaccination card and/or ink-marked finger) and reasons for non-vaccination in this SIA. Local data collectors who conducted pre-SIA survey were used for interviews after a refresher training. Data analysis was performed using Microsoft Excel and OpenEpi. Vaccination coverage was calculated as the proportion of children that reported receiving the vaccine in the survey sample divided by the total number of children in the target vaccination age in the survey sample with respective 95% confidence intervals (95%CI).

**Ethical approval**

The results described in this paper are purely observational and were done as part of monitoring, implementation and evaluation of routine activities in the MSF project in Kobanê, Syria. The study was conducted with permission from the Medical Director of MSF-OCA and exempted from ethical review by The MSF Ethics Review Board.

## Results

**Pre-SIA Vaccine Coverage Survey**

The survey was implemented in June 2015 and included 212 randomly selected children under 5 years of age from 135 households (100% response rate; 1 household contained no children under 5 years). The mean age of the children was 27 months (median age: 25 months; interquartile range: 13-42 months) and 49.1% (n=104) were female. Fifty three children (25.0%; 95%=18.8-32.4%) had a vaccination card available.

Of the 212 children, 168 (79.3%; 95%CI=72.7-84.6%) had received one vaccine or more in their lifetime and 43 (20.3%; 95%CI: 15.1-26.6%) had received all vaccines due by their age. Of the 43 fully vaccinated children, only one was under 12 months old; this child had received all their vaccinations outside of Syria.

Table 1 shows overall vaccination coverage for the main EPI antigens among children under five years in Kobanê; Table 2 shows vaccination coverage for these antigens by age sub-group.

**Table 1: Coverage of EPI schedule vaccinations in children under 5 years in Kobanê, northern Syria, 2015:** NB: DTP=Diptheria+Tetanus+Pertussis; MMR= Measles + Mumps + Rubella; Time-lags are explained in the methods section; *According to Government of Syria EPI schedule used in Kobanê until vaccination ceased in 2014; ~Defined as those who have completed the month when vaccine is due, i.e. if vaccine is due at 3 months, includes all children 4 months and older; **Polio 1 and 2 were IPV (inactivated polio vaccine) and Polio 3 was OPV (oral polio vaccine).

Antigen	Vaccination	Age at which vaccine should be administered*	Number of children eligible to have received vaccine (according to age)~	Number who received vaccine	Vaccination coverage [% (95%CI)]
BCG	BCG	Birth (within first month of life)	207	127	61.3 (53.7-68.4)
DTP	DTP1	3 months	198	107	54.0 (46.1-61.8)
	DTP 2	4 months	196	77	39.3 (31.9-47.2)
	DTP 3	5 months	193	52	26.9 (20.5-34.5)
Polio**	Polio 1	3 months	198	144	72.7 (65.3-79.1)
	Polio 2	4 months	196	108	55.1 (47.5-62.4)
	Polio 3	5 months	193	71	36.8 (29.5-44.7)
MMR	MMR 1	12 months	160	55	34.4 (26.9-42.7)

**Table 2: Coverage of EPI schedule vaccinations by age sub-group in children under 5 years in Kobanê, northern Syria, 2015.** 

		Vaccination coverage by age group % (95%CI; proportion)				
Antigen	Vaccination	0-11 Months	12-23 Months	24-35 Months	36-47 Months	48-59 Months
BCG	BCG	19.6 (10.6-33.3; 10/51)	63.3 (47.8-76.4; 31/49)	70.6 (51.9-84.2; 24/34)	84.1 (69.3-92.5; 37/44)	73.5 (55.2-86.2; 25/34)
DTP & Hib	DTP 1/Hib 1	9.8 (4.0-22.1; 5/51)	53.1 (38.2-67.4; 26/49)	64.7 (46.0-79.8; 22/34)	70.5 (53.8-83.0; 31/44)	67.7 (49.2-81.9; 23/34
	DTP 2/Hib 2	5.9 (1.8-17.3; 3/51)	38.8 (25.4-54.1; 19/49)	38.2 (23.1-56.1; 13/34)	47.7 (32.4-63.5; 21/44)	61.8 (43.4-77.2; 21/34)
	DTP 3/Hib 3	2.0 (0.2-13.4; 1/51)	12.2 (5.4-25.4; 6/49)	14.7 (5.9-32.2; 5/34)	45.5 (30.4-61.4; 20/44)	58.8 (40.6-74.9; 20/34)
Polio	Polio 1	21.6 (12.1-35.5; 11/51)	73.5 (58.7-84.4; 36/49)	88.2 (71.2-95.8; 30/34)	86.4 (71.9-94.0; 38/44)	85.3 (67.9-94.1; 29/34)
	Polio 2	9.8 (4.0-22.1; 5/51)	42.8 (28.9-58.0; 21/49)	64.7 (47.7-78.7; 22/34)	75.0 (58.3-86.6; 33/44)	79.4 (61.4-90.3; 27/34)
	Polio 3	7.8 (2.9-19.7; 4/51)	22.5 (12.5-36.9; 11/49)	35.3 (20.2-54.0; 12/34)	52.3 (36.5-67.6; 23/44)	61.7 (43.4-77.3; 21/34)
MMR	MMR 1	-	8.2 (3.0-20.5; 4/49)	29.4 (15.6-48.4; 10/34)	50.0 (34.4-65.6; 22/44)	55.9 (38.8-71.7; 19/34)
Fully vaccinated for age		2.0 (0.3-13.5; 1/51)	4.1 (1.0-15.6; 2/49)	8.8 (2.7-25.4; 3/34)	40.9 (26.4-57.2; 18/44)	55.9 (38.8-71.7; 19/34)

Sixty eight of the 212 children (32.1%; 95%CI: 25.4-39.6%) had received one or more doses of polio vaccine as part of a mass vaccination campaign (whether in Syria, or as a refugee in Turkey or Iraq). Of the 209 children who spent some time as a refugee in Turkey or Iraq as a result of the conflict in Kobanê, 47 (22.5%; 95%CI: 16.8-29.4%) received one or more vaccines whilst a refugee. Fifty four out of 212 children (25.5%; 95%CI: 19.8-31.9%) received more than 3 polio doses (between 4 and 7 doses in total) most likely during polio SIAs.

Coverage rates for vaccinations were not different between female and male children, for instance, coverage of the DTP1 vaccine was 50.9% for males (95%CI: 41.1-60.7%) and 50.0% for females (95% CI: 40.0%-0.0%), and for Polio3 was 32.4% for males (95%CI: 23.7-42.1%) and 34.6% for females (95%CI: 25.6-44.6%).

Reasons for not vaccinating were given by 107 of the 111 parents/guardians of incompletely vaccinated children. The most commonly cited reasons were: vaccination not available (when in Syria) (39.6%; 95%CI: 30.9-49.0%); vaccination not available (when a refugee) (14.4%; 95%CI: 8.8-21.9%); child was ill at the time of vaccination (11.7%; 95%CI: 6.7-18.7%); and because they did not know the place or time for vaccination (when a refugee) (9.0%; 95%CI: 4.7-15.5%).

**VPD Risk Analysis**

The first step of the MSF VPD risk analysis (assessing contextual risk factors) suggested that measles, cholera, pneumococcal disease and Haemophilus influenzae (Hib) pneumonia were the VPDs with the greatest spread likelihood in this context, given the concerning (but not serious) state of water/hygiene/sanitation, low access to health care and high birth and chronic disease rates (see Table 3).

**Table 3: Results of the first step (contextual risk factor assessment) of the MSF VPD Risk Analysis for Kobanê, April 2015.** * These diseases are usually included in the EPI; PCV = pneumococcal vaccine; WHS = Water, hygiene and sanitation

		Overcrowding	Malnutrition	Poor WHS	Low Access	High birthrate	High chronic disease	Spread likelihood
Epidemic prone	Measles*	0	0	2	4	4	2	12
	Cholera	0	0	4	4	1	1	10
	Polio*	0	0	4	1	1	1	7
	Diphtheria*	0	0	1	2	1	1	5
	Pertussis*	0	0	1	2	4	1	8
Burden	PCV	0	0	1	4	4	4	13
	Hib*	0	0	2	4	4	2	12
Region/ Season	Typhoid	0	0	4	2	1	2	9
	Meningitis A	0	0	1	4	1	2	8
	Yellow Fever	0	0	2	1	1	1	5
	Japanes Encephalitis	0	?	2	2	2	1	7

The second step of the analysis (assessing aggravating risk factors) highlighted the limited data available on health and vaccination coverage for this area since the conflict began. However, available anecdotal evidence highlighted: low vaccine coverage levels for all antigens; reported cases of measles polio, typhoid and Hepatitis A in the country; and very high levels of respiratory tract infection in the area.

Taking into account the findings of steps one and two, and contextual restrictions (e.g. current absence of EPI vaccination; that prevention of/response to a waterborne disease outbreak would focus on water and sanitation measures before vaccination), it was concluded that the most important vaccinations to consider were measles, Hib and PCV. The decision to not conduct SIAs for PCV was based on the fact that this antigen was not currently approved under the Syrian Ministry of Health guidelines for the Extended Programme for Immunisation (EPI) and thus could not be imported into the country. The decision to not conduct a mass vaccination campaign for Hib was based on the practical considerations that there were not enough supplies on the ground at the time to be able to achieve an acceptable level of coverage. However, pentavalent vaccination (which includes Hib) was immediately installed as part of the EPI activities within MSF health facilities. Based on the low coverage from the vaccination coverage survey for measles, and the above-mentioned considerations, MSF recommended that a measles (and polio) mass vaccination campaign be undertaken. The polio vaccination remained under the coordination of other international stakeholders (United Nations Children’s Fund (UNICEF) and the WHO).

**Measles SIA**

During the measles SIA in August 2015, tally sheet records indicated that a total of 3410 children aged 6-59 months were vaccinated. This gives an administrative vaccination coverage of 71.0%. Of these 3410 children, 1637 (48.0%) were female and 290 (9.8%) resided outside of Kobanê city (SIA activities were carried out by the KHA in the surrounding canton in the following days). Two cases of fever after vaccination were reported which were symptomatically managed and followed up. No other adverse effects following immunization were reported. Observations on the challenges faced in implementing this SIA were made by MSF staff and are considered in the Discussion section below.

**Post-SIA Vaccine Coverage Survey**

In September 2015, 282 households containing 306 children aged 6-59 months were included in the post SIA vaccine coverage survey. The response rate was 100%. The mean age was 30 months (median age: 24 months; interquartile range: 12-48 months); 49.7% (n=152) were female. Two hundred and eighty children were included in the final analysis as 26 children had returned to Kobanê after the SIA. Of these 280 children, 229 were reported to have received a measles vaccination in the SIA, giving a coverage of 81.8% (95%CI: 76.9-85.9%). An SIA vaccination card was available for 94.8% (n=217); 8 children (3.5%) still had a visible ink marking on the left fifth finger. The most common reason for non-vaccination amongst the 51 children who had not been vaccinated was that the caretaker was busy during the campaign (n=22, 43.1%, 95%CI: 30.1-56.9%). The next most common reasons were that the caretaker did not know about the vaccination campaign and that the child was sick during the vaccination campaign (n=7, 13.7% each; 95%CI: 6.2-25.3%).

## Discussion

The impact of the Syrian crisis on public health in the country is evident from these results: in Kobanê, the vaccination coverage for all vaccine antigens included in the EPI schedule of Syria was poor with only 1 in 5 (20.3%) children under 5 years demonstrating complete vaccination coverage for their age.. In fact, only one out of 51 children (2.0%) less than 12 months of age was fully vaccinated for age, as compared to 19 out of 34 (55.9%) in the 48-59 month age group, highlighting the fact that younger children were more severely affected by a progressively crippled health system. Compared to data from 2010 from the Syrian Ministry of Health where greater than 80% of the target age group was reported to be vaccinated the difference in coverage rates is striking 15. The results highlight beyond any doubt how the collapse of the functioning health system during the current conflict is impacting children 16.

The low rates of MMR, polio, PCV and Hib coverage in all children under 5 years of age in Kobanê illustrate the high level of susceptibility that children have towards these diseases which we know can have serious medical consequences ranging from pneumonia to respiratory failure and sometimes death.

The choice by MSF to recommend a measles and polio vaccination campaign in Kobanê following the return of a large proportion of the population in 2015 was based on the low vaccination coverage rates for polio and MMR in the returning population. However, it also took into consideration a careful risk assessment around VPDs in Kobanê at that time. In difficult humanitarian conditions, the possible impact of a measles outbreak on a vulnerable population in Kobanê could have been disastrous and was thus prioritised. Polio vaccination was already being planned by other partners with the support of the World Health Organisation (WHO). Other important VPDs highlighted in this risk assessment like PCV were de-prioritised as they were not included in the official EPI schedule of the Syrian Ministry of Health and thus could not be imported into the country within the available time window. These were therefore put forward as recommendations by MSF to the KHA to include these antigens in the EPI programme of Kobanê as soon as it was operational again.

The strength of the VPD risk assessment tool is that it is a rapid, comprehensive and easy to use tool that provides an approach for deciding which vaccines, if pre-emptively and properly delivered at the outset of an emergency would constitute high priority public-health interventions and would reduce avoidable death and disease. Our inability to deliver pneumococcal vaccination in Northern Syria (despite being prioritised by use of the tool) as it wasn’t yet approved in the Syrian immunisation schedule is an example of local practical implementation difficulties that are not taken into account by the tool.

Maintaining adequate vaccination coverage levels in any population is difficult without a well-functioning EPI system. In many countries, the use of SIAs is commonly employed in order to catch up with vaccination coverage for target diseases in a rapid way. However, during active conflict and therefore a continuous flux of displaced populations, maintaining the immunity levels will remain a challenge even with the SIA approach.

All public health stakeholders in Kobanê agreed that the SIA for measles was important and that there was a small window of opportunity present to implement it. However, hesitance remained as a result of the 2014 tragedy in Idlib city, Syria, where 15 children died when an error was made with the measles vaccine diluent and the measles vaccination campaign had to be stopped prematurely 17. The SIA in Kobanê was therefore the first time (to our knowledge) since that event that a measles vaccination campaign was implemented in the region.

The exodus of qualified health workers from Syria since the war began meant that the few left behind had to take on additional responsibilities beyond their expertise or experience^3^. Repeated attacks on health structures across the country in defiance of the tenets of the Geneva Conventions had left MSF with no alternative other than to have limited or no international staff presence in their field locations 18. This provided practical challenges in training, supporting, implementing and monitoring a SIA and placed an increased burden on the remaining health care system.

There was also the possibility of terror attacks on fixed vaccination sites that had to be recognised and a contingency plan put into place. To circumvent this, doing measles vaccination from house to house was also discussed but considered not feasible at the time. It was then decided that four decentralised sites across Kobanê town would be established and vaccination run over 5 days to avoid large crowds at any given time.

The need for surveillance and early warning systems for outbreaks of VPDs – including those using non-formal reporting channels –is increasingly important. Only with rapid alerting of suspected cases and clusters of measles, polio and other VPDs can response measures to mitigate their impact be implemented. Adequate response would then require the ability to transport vaccines, medical supplies (syringes, cooling boxes etc.) and other healthcare related supplies into northern Syria; something which has been impossible for the majority of 2016 due to near-complete border closures.

In the post-vaccination survey, 8.5% of those sampled had not been present in Kobanê at the time of the SIA, again highlighting the fact that, unless routine immunization services are restored, maintaining an acceptable level of measles coverage will be very challenging when a significant proportion of the population is mobile due to insecurity. Despite not achieving the targeted 95% coverage that we usually aim for in emergency settings, the result from the SIA was encouraging when taking into considerations the contextual challenges for its implementation.

The post-SIA coverage of 82% differed from that expected by administering measles vaccine to 3410 of 4800 (71%) children. In emergency situations it is common place to conduct post vaccination coverage surveys to have a more accurate understanding of the vaccination coverage levels achieved during the vaccination campaign as population numbers used to plan such campaigns are often flawed due to significant population movements.

The use of GPS based sampling for surveys in emergency contexts is not uncommon and has been used effectively in insecure environments or where traditional census data is absent to use as a sampling frame for random household selected 19. The possible limitation around this method is that it favours households located in sparsely populated areas compared to densely populated areas. We do not think this potential bias affected the pre and post SIA significantly as Kobane town is very small and we assumed that the population was therefore homogeneously distributed within the boundaries of the town that we generated.

While in this manuscript, we describe an SIA that MSF managed to conduct, the situation in Syria does indeed limit the use of SIAs primarily in two ways. 1. SIAs are much delayed in their implementation due to security and logistic constraints and one has to wait for a window of opportunity in the form of a favorable environment to conduct the SIA. 2. The geographical area in which SIAs are conducted is much smaller than what is needed and often occur in relatively safer areas closer to the international borders with neighboring countries rather than deeper into Syrian territory where the armed conflict may be more active.

## Conclusions

Access to EPI vaccination for children is a key example of preventative public health interventions that have been curtailed in Northern Syria since the start of the conflict.

These findings demonstrate that collapse of the formal public health system has led to an increasingly large group of children who are susceptible to infectious diseases with serious consequences, with younger children most vulnerable.

We call on all health actors and the international community to work towards re-establishment of EPI activities as a priority to ensure that children who have had no access to vaccination in the last five years are adequately protected for VPDs as soon as possible.

## Data Sharing

Data are available on request in accordance with MSF's data sharing policy due to the sensitivity of the data. Requests for access to data should be made to data.sharing@msf.org. For more information please see:

MSF’s Data Sharing Policy: http://fieldresearch.msf.org/msf/handle/10144/306501

## Funding

Médecins Sans Frontières – Operational Centre Amsterdam (MSF-OCA) funded the humanitarian response activity described in this manuscript. MSF-OCA had no role in the study design, data analysis, decision to publish or preparation of the manuscript. The authors received no specific funding for this work.

## Corresponding Author

Alan de Lima Pereira: dr.a.d.pereira@gmail.com

Communications can also be addressed to Annick Lenglet: annick.lenglet@amsterdam.msf.org.

## Competing Interests

The authors have declared that no competing interests exist.
